# Knowledge distillation based on multi-layer fusion features

**DOI:** 10.1371/journal.pone.0285901

**Published:** 2023-08-28

**Authors:** Shengyuan Tan, Rongzuo Guo, Jialiang Tang, Ning Jiang, Junying Zou

**Affiliations:** 1 College of Computer Science, Sichuan Normal Univeersity, Chengdu, Sichuan, 610101, China; 2 School of Computer Science and Technology, Southwest University of Science and Technology, Mianyang, Sichuan, 621010, China; Zhejiang University of Technology, CHINA

## Abstract

Knowledge distillation improves the performance of a small student network by promoting it to learn the knowledge from a pre-trained high-performance but bulky teacher network. Generally, most of the current knowledge distillation methods extract relatively simple features from the middle or bottom layer of teacher network for knowledge transmission. However, the above methods ignore the fusion of features, and the fused features contain richer information. We believe that the richer and better information contained in the knowledge delivered by teachers to students, the easier it is for students to perform better. In this paper, we propose a new method called Multi-feature Fusion Knowledge Distillation (MFKD) to extract and utilize the expressive fusion features of teacher network. Specifically, we extract feature maps from different positions of the network, i.e., the middle layer, the bottom layer, and even the front layer of the network. To properly utilize these features, this method designs a multi-feature fusion scheme to integrate them together. Compared to features extracted from single location of teacher network, the final fusion feature map contains meaningful information. Extensive experiments on image classification tasks demonstrate that the student network trained by our MFKD can learn from the fusion features, leading to superior performance. The results show that MFKD can improve the Top-1 accuracy of ResNet20 and VGG8 by 1.82% and 3.35% respectively on the CIFAR-100 dataset, which is better than state-of-the-art many existing methods.

## Introduction

The great success of computer vision in the past few decades is inseparable from deep neural network (DNN) [1–4] due to the reason that DNN can show excellent performance on many vision tasks [5,6]. Generally speaking, the performance of a network model is positively related to the amount of parameters and computation of the model. However, it is unable to deploy a model with a large amount of parameters on embedded devices with limited resources. The existing methods to solve this problem mainly include knowledge distillation (KD) [7,8], network pruning [9,10], network quantization [11,12] and low-rank factorization [13], among which KD is a very effective method.

**Fig 1:**
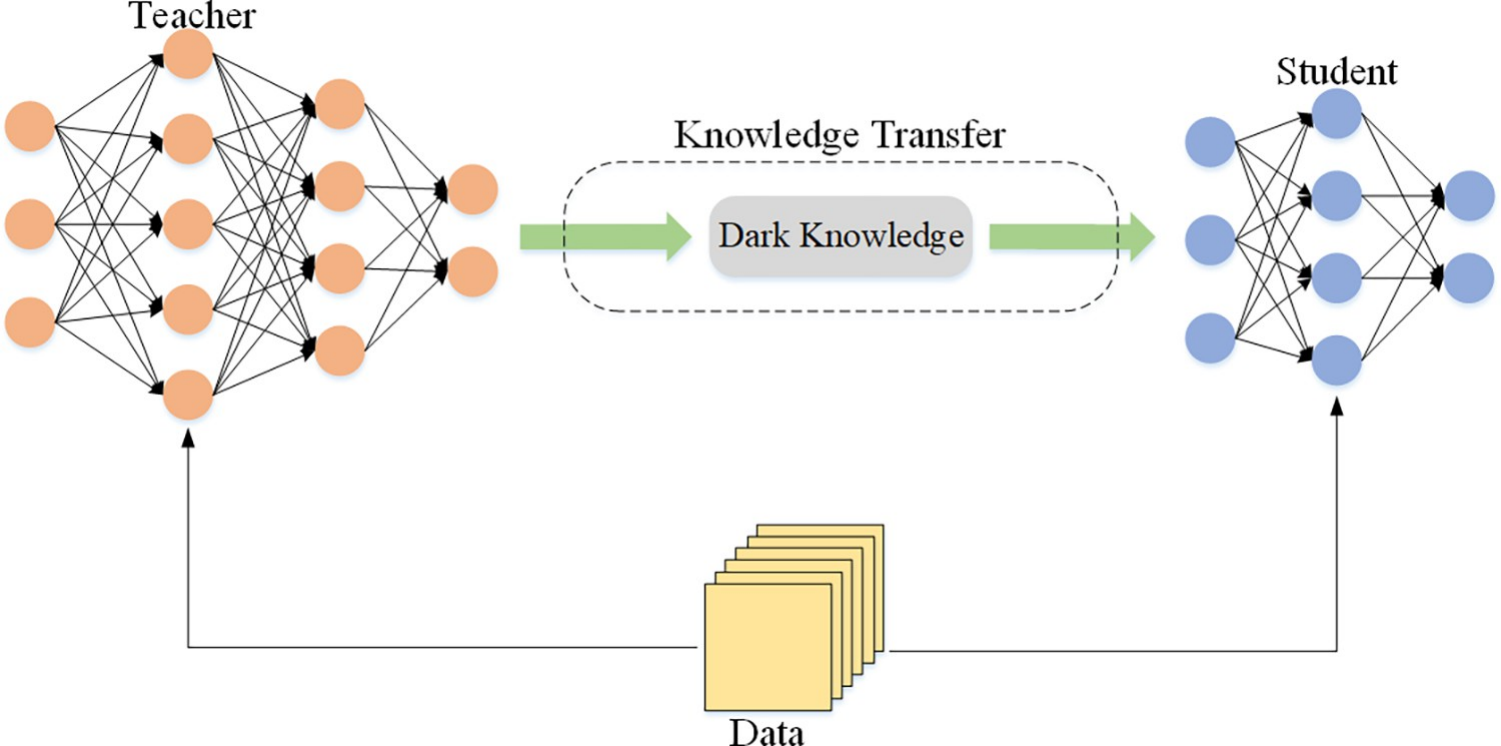
Schematic diagram of the essence of knowledge distillation (KD).

The essence of KD is to enable the small student network with a small number of parameters to learn the “dark knowledge” taught by the large teacher network with a large number of parameters, Fig 1 shows the essence of KD The student network can achieve considerable performance improvement, and even make its performance close to that of the teacher network. In the existing KD methods, there are two main ways for “dark knowledge” transfer including logtis distillation [7,14] and features distillation [15,16]. Logits distillation performs knowledge transfer by minimizing the relative entropy of *logits* predicted by teachers and students. Compared with logits distillation, the features-based methods have a good performance on many tasks, such as the model compression task of image classification network and the model compression task of object detection network [17,18]. Therefore, researchers are more inclined to the features distillation in recent years. But most of them ignored the fact that in the same neural network, the feature maps generated by different neural network layers located in different locations contained different information which may yield different results when they are applied to KD.

It is a known fact that the resolution of feature maps generated by neural network will change from high to low under the action of downsampling with layer by layer computation of neural network. And low resolution feature maps have stronger semantic information, while high resolution feature maps have more accurate localization activation due to fewer downsampling times [19]. Thus, we believe that extracting the feature maps of different layers from the neural network for fusion and then distillation can make the knowledge taught by the teacher to the student more high-quality, thereby improving the learning effect of the students.

In the present work, a new method called *Multi-feature Fusion Knowledge Distillation* (MFKD) is proposed. First, we extract the feature maps generated from different layers of teacher network, and employ a feature pyramid to fuse the extracted feature maps. During this process, the feature maps are corrected. We then obtain the fusion features from the student network by the same way. Finally, the mean squared error (MSE) between the fusion feature maps produced by the teacher and the student is minimized for promoting knowledge transfer.

In a summary, our main contributions are summarized as follows:

- Considering that fused features are rich in information, we perform knowledge transfer by fusing feature maps generated by multiple neural network layers.
- In the process of fusion, attention mechanism is added to correct the feature map so as to obtain better multi-layer fusion features.
- For different datasets and different networks, the distillation effect can be significantly improved.

### Related work

Since this paper focuses on compressing neural network models by the method of KD, some excellent related work published in recent years on the model compression and KD are given below.

#### a) Model Compression

In order to solve the problem of deploying DNN models on embedded devices with limited storage and computing resources, model compression technology is proposed. There are several methods to realize model compression: network pruning, network quantization, low-rank factorization and KD. Since there are a large number of redundant parameters in the neural network models, and these parameters have a very subtle impact on the final result of the network model. The main idea of pruning is to cut out the unimportant neurons and filters in the network to achieve the purpose of compressing the model [20,21]. The purpose of quantization is to replace the high-precision numbers stored in the original model with low precision numbers [22]. Low-rank factorization refers to sparse the convolution kernel matrix by combining dimensions and imposing low-rank constraints [23]. The most of weight vectors are distributed in the low-rank subspace, and a small number of basis vectors can be used to reconstruct the convolution kernel matrix to achieve the purpose of reducing storage space.

#### b) Knowledge Distillation

The framework of knowledge distillation was first proposed by Hinton et al. [7] which is based on logits. By introducing the concept of temperature (*T*), logits generated by teacher model are softened to obtain soft targets, and logits generated by student are then used to simulate soft targets. However, most of the current knowledge distillation methods are based on features. According to the extraction location of features, we have the following divisions:

#### (b1) Middle layer

Romero et al. [15] proposed a two-stage method which extracts the respective intermediate layer features in the teacher network and the student network, and allows the student features to fit the teacher features to obtain a pre-trained student model in the first stage. In the second stage, with the help of parameters obtained in the first stage, the authors used soft targets to train to get the complete student model parameters.

#### (b2) Bottom layer

*Factor Transfer* **(**FT) [24] is distilled at the end of the last layer group. Here the convolution layer group refers to the combination of *N* convolution layers with the same size of the output feature map. For example, ResNet56 has three layer groups with nine convolutional layers in each layer group. The method of Heo et al. [25] is slightly different with FT. They chose the position of extracting features between the first ReLU and the end of the layer group which can better preserve the information conveyed by the teacher. *Contrastive Representation Distillation* (CRD) [17] adds the method of contrastive learning to knowledge distillation, and transfers knowledge by comparing the penultimate layer features (before logits) of the teacher and student networks.

#### (b3) Multilayer

Different from FitNets extracting the output features of any intermediate layer, AT [26], FSP [27] and Jacobian [28] methods extract the output features of each convolution layer group when the teacher network and the student network have different depths. Chen et al. [16] used the multi-layer features in the teacher network to guide the learning of a certain layer in the student network to realize the transfer of knowledge in different layers.

It differs from the aforementioned methods, we not only extract the feature maps of different layers from the neural network, but also fuse these feature maps to obtain fused feature maps containing abundant information and having good representation ability. Thereby, it is convenient for students to learn on the network and improve the effect of KD.

### Our method

Multi-feature Fusion Knowledge Distillation (MFKD) is a feature-based distillation method. So, for MFKD, feature extraction and processing are particularly important. In this section, the details of the MFKD including notation, features extraction and correction, feature fusion pyramid, and hyper-parameter *p* are introduced.

#### • Notation

Supposed that the teacher network and the student network are represented by *N*^*t*^ and *N*^*s*^ respectively, the convolution network part of *N*^*t*^ has *i* layer groups, and *N*^*s*^ has *j* layer groups. The input is represented by *x*. When *x* is processed by the network, the set of output feature maps obtained by each layer group in *N*^*t*^ can be expressed as $O^{t}=\{o_{1}^{t},o_{2}^{t},o_{3}^{t},\ldots ,\;o_{i}^{t}\}$ in which $o_{k}^{t}$ is the output of the *k*th layer group in *N*^*t*^, and is also the input of the (*k*+1)th layer group. Similarly, the set of output feature maps obtained by each layer group in *N*^*s*^ can be expressed as $O^{s}=\{o_{1}^{s},o_{2}^{s},o_{3}^{s},\ldots ,\;o_{j}^{s}\}$. Among them, $o_{i}^{t}\;and\;o_{j}^{s}$ are also respectively the final output of the entire convolutional layer in *N*^*t*^ and *N*^*s*^. We select *m* feature maps from *O*^*t*^ to form a feature extraction set of *N*^*t*^, which ${\bar{O}}^{t}=\{{\bar{o}}_{1}^{t},{\bar{o}}_{2}^{t},{\bar{o}}_{3}^{t},\ldots ,\;{\bar{o}}_{m}^{t}\}(m\leq i,\;{\bar{O}}^{t}\subseteq O^{t})$, the resolution of ${\bar{o}}_{l}^{t}$ is higher than ${\bar{o}}_{l-1}^{t}(1<l\leq m)$. The same ${\bar{O}}^{s}$ represents the feature extraction set of *N*^*s*^ with *n* feature maps ($n\leq j,\;{\bar{O}}^{s}\subseteq O^{s}$). The final fusion features of *N*^*t*^ and *N*^*s*^ are denoted by $F_{m-1}^{t}$ and $F_{n-1}^{s}$, respectively.

Therefore, the knowledge transfer between teachers and students can be described as an optimization problem with the following expression:

$$minMSE(F_{n-1}^{s},F_{m-1}^{t})$$ (1)

#### • Features extraction and correction

**Fig 2:**
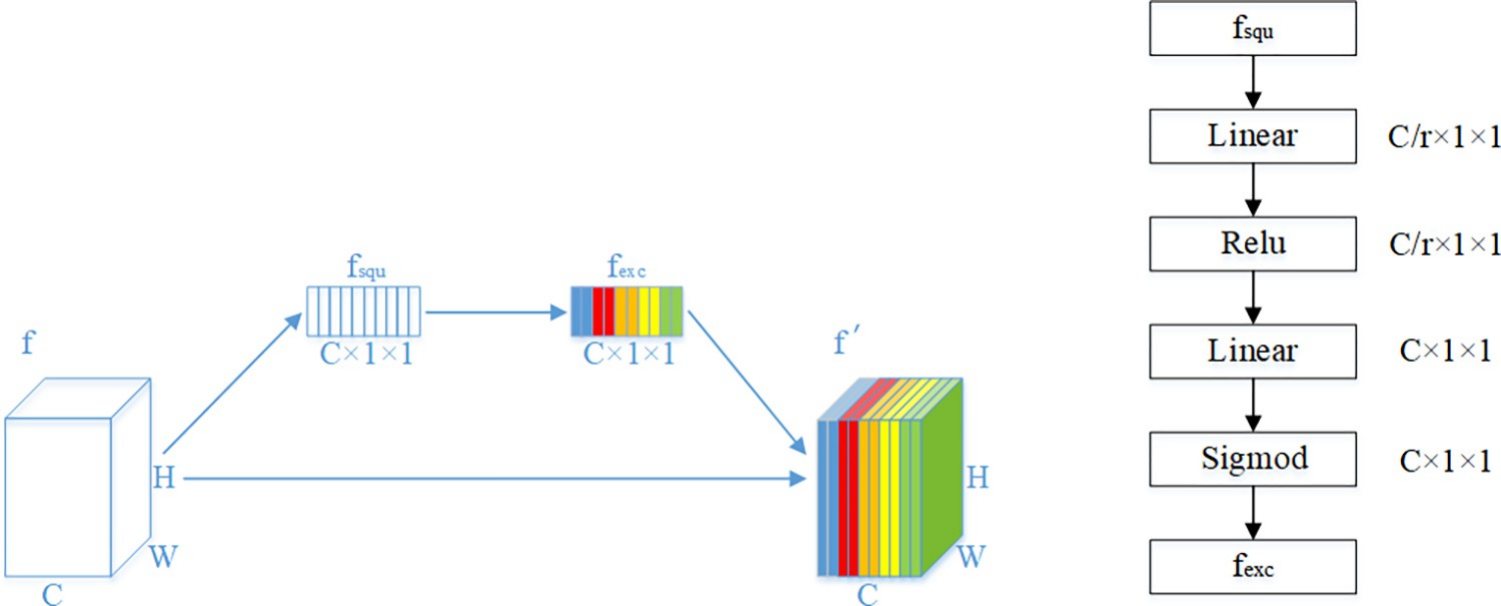
The framework of the Squeeze-and-Excitation block.

We extract features at different positions in the teacher network and the student network to obtain sets ${\bar{O}}^{t}$ and ${\bar{O}}^{s}$, and use the attention mechanism to correct all feature maps in the set. The theory of the Squeeze-and-Excitation block (SEblock) [29] is used in this paper to achieve the purpose of feature correction, and the framework of the theory is displayed in the following Fig 2.

In Fig 1(A), SEblock consists of two parts: squeeze and excitation. In the squeeze phase, global average pooling is used for converting the input feature map *f* with size *C*×*H*×*W* into the output feature map *f*_*squ*_ with size *C*×1×1, and compressing the information in *f* into *f*_*squ*_. In the excitation phase, the mainly idea is a simple gating mechanism with sigmoid activation. This gating mechanism is parameterized by two fully connected layers with a dimensionality reduction ratio of *r* (in this paper, *r* = 16). Finally, performing the channel multiplication of *f* and *f*_*exc*_ to complete the mapping from *f* to *f*′, and the mathematical expression is given by

$$f^{\prime }=f\cdot f_{exc}.$$ (2)

#### • Feature fusion pyramid

Since the size of the feature maps generated by different locations of the network is different, it impossible to directly fuse the features. Therefore, we use the feature fusion pyramid method to fuse features. The feature fusion pyramid structure is based on the pyramid feature hierarchy of convolutional neural network [1,2], with the purpose of fusing high-level semantic information and low-level localization features in the neural network, and performing knowledge transfer.

**Fig 3:**
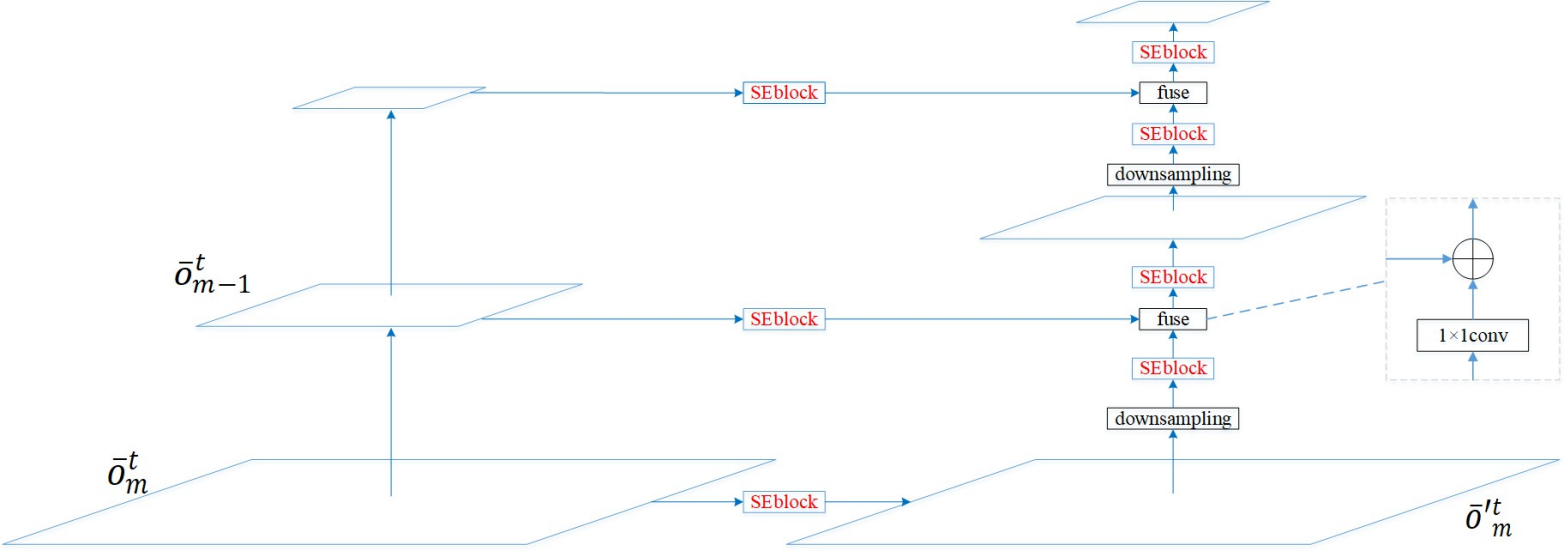
Feature fusion pyramid of an *N*^*t*^.

Taking *N*^*t*^ as an example, the framework of the feature fusion pyramid is shown in Fig 3. We first correct ${\bar{o}}_{m}^{t}$ and ${\bar{o}}_{m-1}^{t}$ to obtain ${{\bar{o}}^{\prime }}_{m}^{t}$ and ${{\bar{o}}^{\prime }}_{m-1}^{t}$ with the help of the Squeeze-and-Excitation block, and then fuse these two corrected feature maps. The formula can be expressed as

$$F_{1}^{t}=Fuse({{\bar{o}}^{\prime }}_{m}^{t},{{\bar{o}}^{\prime }}_{m-1}^{t})$$ (3)

Owing to the resolution of ${o^{\prime }}_{m}^{t}$ is higher than ${o^{\prime }}_{m-1}^{t}$, it is necessary to downsample ${o^{\prime }}_{m}^{t}$ before fusion. If element-wise addition is used as the feature fusion method (the ablation study case in experiment section), ${o^{\prime }}_{m}^{t}$ and ${o^{\prime }}_{m-1}^{t}$ should also have the same number of channels, this paper uses 1×1 convolutional layers to increase/decrease the dimension of feature maps. It should be noted that when the feature map is processed by downsampling, dimensionality increase/reduction, and fusion, it will be corrected. It found that the feature correction keeps the information carried by the feature map correct, and the final fusion features can perform well after repeated adjustment.

Actually, the feature map will change after the convolution calculation, and the original information in feature map will also be affected. Therefore, we increase/reduce the dimension of ${{\bar{o}}^{\prime }}_{m}^{t}$ after downsampling so that the dimension of ${{\bar{o}}^{\prime }}_{m}^{t}$ matches that of ${{\bar{o}}^{\prime }}_{m-1}^{t}$. The biggest advantage of this manner is not change the original dimension of ${{\bar{o}}^{\prime }}_{m-1}^{t}$, which can preserve the originality of the information in ${{\bar{o}}^{\prime }}_{m-1}^{t}$.

We next correct $F_{1}^{t}$ and ${\bar{o}}_{m-2}^{t}$ to get ${F^{\prime }}_{1}^{t}$ and ${{\bar{o}}^{\prime }}_{m-2}^{t}$, and then fuse them. Similar to the same argument of ${{\bar{o}}^{\prime }}_{m}^{t}$ and ${{\bar{o}}^{\prime }}_{m-1}^{t}$, both downsampling and dimension up/down processing are applied to ${F^{\prime }}_{1}^{t}$. Therefore, after *m*-1 times of fusion, we will get the final fusion features of *N*^*t*^, which can be expressed as

$$F_{m-1}^{t}=Fuse({F^{\prime }}_{m-2}^{t},{{\bar{o}}^{\prime }}_{1}^{t}).$$ (4)

In the same way, the final fusion feature $F_{n-1}^{s}$ of *N*^*s*^ can be obtained. For MFKD, the realization of knowledge transfer is to let $F_{n-1}^{s}$ simulate $F_{m-1}^{t}$, and short the distance between them. This problem is described in Eq 1.

#### • Hyperparameter *p*

To further improve the performance of MFKD, a hyperparameter *p* is introduced. In the process of *N*^*s*^ learning from *N*^*t*^, when the prediction results of *N*^*t*^ are good, the *N*^*s*^ should be to learn from *N*^*t*^; when the prediction results of *N*^*t*^ are bad, the *N*^*s*^ should be to learn from ground-truth labels. In this way, *p* becomes the criterion for judging the quality of prediction results of *N*^*t*^. So the rest task is to set the optimal values of *p*. Here we divide a dataset into *α* batches, and send them to *N*^*t*^ to calculate the prediction results. Using the ascending order, these results are arranged in a vector *PRE* = {*pre*_1_, *pre*_2_, *pre*_3_,…,*pre*_*α*_} where *pre*_*i*_<*pre*_*j*_ when *i*<*j*. Furthermore, we set a percentage *β* to determine the value of *p* = *pre*_*α*_·*β*.

## Experiment

### • Implement Details

#### a) Dataset

Two classical image classification datasets, CIFAR-10 [30] and CIFAR-100 [30], are selected to validate the effectiveness of MFKD. The details of the two datasets are described in the following.

CIFAR-10 has a total of 60K color images, including a training set with 50K images and a test set with 10K images. Each image size is 32 × 32pixels. There are 10 categories in total, and each category has 6K images.

Similar to the CIFAR-10, CIFAR-100 has also 60K color images including 50K training images and 10K test images. Further, it has a total of 100 categories where each category has 600 images including 500 training images and 100 test images. The image size is 32× 32 pixels.

#### b) Models

Three kinds of neural networks are applied in our experiment including: ResNet [4] which is narrow and deep; WideResNet [31] which is wider but shallower than ResNet; VGG [2] which is a classical linear structure network. In addition, our experiments focus on knowledge distillation between networks with the same architectural style, such as: teacher is VGG13, student is VGG8.

#### c) Setting

**Data augmentation**. For the training set of CIFAR dataset, we first fill 4pixels around the image, then randomly cut the image to 32×32pixels, perform random horizontal flip with a probability of 0.5, and finally normalize the image with the mean and standard deviation of each channel. But for the test datasets, the normalized is only applied to process data.

**Training parameter settings (Table 1)**. To verify the effectiveness of MFKD, we use the same parameter settings for baseline training and distillation training. The Stochastic Gradient Descent (SGD) algorithm is applied for network optimization where the momentum of SGD is set to 0.9 and the weight decay is 5e-4. The initial learning rate is 0.05, and it decays to 0.1 times of the previous time at the 150th, 180th, and 210th epochs, respectively. A total of 240 trained epochs are predetermined, and the batchsize at training time is 64.

**Table 1:** Details of experimental parameter settings.

padding	crop	flip	lr	lr_decay	momentum	weight_decay	batch_size	epoch
4	32	0.5	0.05	0.1	0.9	5e-4	64	240

### • Results

#### a) CIFAR-10

In the CIFAR-10 dataset, we perform two groups of cases, one is ResNet56 as teacher, ResNet20 as student; the other is WRN_40_2 as teacher, WRN_16_2 as student.

In the first case, both ResNet56 and ResNet20 contain three convolutional layer groups, we extract features at the output position of each layer group, and use the average of three experimental results as the final result. Compared to conventional training, MFKD improves the Top-1 accuracy of ResNet20 by 0.48%, which is also slightly better than several other methods. The experimental results are shown in Table 2.

**Table 2:** Top-1 accuracy of student network with ResNet20 on CIFAR-10 test dataset.

Model	FLOPS	Params	Memory	Top-1 Accuracy
Teacher: ResNet56	~126.84M	~0.86M	~5.09MB	93.90%
Student: ResNet20 (beaseline)	~41.21M	~0.27M	~1.81MB	92.49%
Student: ResNet20 (KD [7])	~41.21M	~0.27M	~1.81MB	92.78%
Student: ResNet20 (Fitnets [15])	~41.21M	~0.27M	~1.81MB	92.55%
Student: ResNet20 (FSP [27])	~41.21M	~0.27M	~1.81MB	91.93%
Student: ResNet20 (NST [32])	~41.21M	~0.27M	~1.81MB	92.81%
Student: ResNet20 (VID [33])	~41.21M	~0.27M	~1.81MB	92.80%
Student: ResNet20 (CCKD [34])	~41.21M	~0.27M	~1.81MB	92.39%
Student: ResNet20 (RKD [35])	~41.21M	~0.27M	~1.81MB	92.71%
Student: ResNet20 (MFKD)	~41.21M	~0.27M	~1.81MB	**92.97%**

In the second case, WRN_40_2 and WRN_16_2 both have three convolution layer groups, and we extract the output feature maps of each layer group for fusion. Taking average of the three experiments as the final result, our method improves the Top-1 accuracy of WRN_16_2 by 0.59% compared with the conventional training, which is also slightly better than the other methods. The experimental results are tabulated in Table 3.

**Table 3:** Top-1 accuracy of student network WRN_16_2 on CIFAR-10 test dataset.

Model	FLOPS	Params	Memory	Top-1 Accuracy
Teacher: WRN_40_2	~329.71M	~2.24M	~8.28MB	94.77%
Student: WRN_16_2 (beaseline)	~101.84M	~0.69M	~3.03MB	93.65%
Student: WRN_16_2 (Fitnets [15])	~101.84M	~0.69M	~3.03MB	93.73%
Student: WRN_16_2 (AT [26])	~101.84M	~0.69M	~3.03MB	94.17%
Student: WRN_16_2 (FSP [27])	~101.84M	~0.69M	~3.03MB	93.43%
Student: WRN_16_2 (NST [32])	~101.84M	~0.69M	~3.03MB	94.15%
Student: WRN_16_2 (SPKD [36])	~101.84M	~0.69M	~3.03MB	94.16%
Student: WRN_16_2 (VID [33])	~101.84M	~0.69M	~3.03MB	94.17%
Student: WRN_16_2 (CCKD [34])	~101.84M	~0.69M	~3.03MB	93.67%
Student: WRN_16_2 (MFKD)	~101.84M	~0.69M	~3.03MB	**94.24%**

#### b) CIFAR-100

For the CIFAR-100 dataset, we consider two groups of cases, the first case is VGG13 as teacher, VGG8 as student; the second case is ResNet56 as teacher, ResNet20 as student.

In case 1, both VGG13 and VGG8 contain five convolutional layer groups, and we select the output positions of the 2nd, the 3rd, and the 4th layer group to extract features to validate MFKD. The average of three experiments is computed as the final experimental result. One checks easily that the MFKD can improve the Top-1 accuracy of VGG8 by 3.35% which compared to one obtained by conventional training. The experimental results are displayed in Table 4.

**Table 4:** Top-1 accuracy of student network with VGG8 on CIFAR-100 test set.

Model	FLOPS	Params	Memory	Top-1 Accuracy
Teacher: VGG13	~285.44M	~9.46M	~1.61MB	74.64%
Student: VGG8 (beaseline)	~96.44M	~3.96M	~0.61MB	70.36%
Student: VGG8 (KD [7])	~96.44M	~3.96M	~0.61MB	72.98%
Student: VGG8 (Fitnets [15])	~96.44M	~3.96M	~0.61MB	71.02%
Student: VGG8 (AT [26])	~96.44M	~3.96M	~0.61MB	71.43%
Student: VGG8 (SPKD [36])	~96.44M	~3.96M	~0.61MB	72.68%
Student: VGG8 (CCKD [34])	~96.44M	~3.96M	~0.61MB	70.71%
Student: VGG8 (VID [33])	~96.44M	~3.96M	~0.61MB	71.23%
Student: VGG8 (RKD [35])	~96.44M	~3.96M	~0.61MB	71.48%
Student: VGG8 (RKT [37])	~96.44M	~3.96M	~0.61MB	72.88%
Student: VGG8 (AB [38])	~96.44M	~3.96M	~0.61MB	70.94%
Student: VGG8 (FT [24])	~96.44M	~3.96M	~0.61MB	70.58%
Student: VGG8 (FSP [27])	~96.44M	~3.96M	~0.61MB	70.23%
Student: VGG8 (NST [32])	~96.44M	~3.96M	~0.61MB	71.53%
Student: VGG8 (MFKD)	~96.44M	~3.96M	~0.61MB	**73.71%**

Similar as the operation scheme of the first case of CIFAR-10, MFKD is carried on the CIFAR-100 dataset. The computational results show that the MFKD improves the Top-1 accuracy of ResNet20 by 1.82% compared to conventional training. The experimental results are given in Table 5.

**Table 5:** Top-1 accuracy of student network with ResNet20 on CIFAR-100 dataset.

Model	FLOPS	Params	Memory	Top-1 Accuracy
Teacher: ResNet56	~126.84M	~0.86M	~5.09MB	72.34%
Student: ResNet20 (beaseline)	~41.21M	~0.27M	~1.81MB	69.06%
Student: ResNet20 (KD [7])	~41.21M	~0.27M	~1.81MB	70.66%
Student: ResNet20 (Fitnets [15])	~41.21M	~0.27M	~1.81MB	69.21%
Student: ResNet20 (AT [26])	~41.21M	~0.27M	~1.81MB	70.55%
Student: ResNet20 (SPKD [36])	~41.21M	~0.27M	~1.81MB	69.67%
Student: ResNet20 (CCKD [34])	~41.21M	~0.27M	~1.81MB	69.63%
Student: ResNet20 (VID [33])	~41.21M	~0.27M	~1.81MB	70.38%
Student: ResNet20 (RKD [35])	~41.21M	~0.27M	~1.81MB	69.61%
Student: ResNet20 (RKT [37])	~41.21M	~0.27M	~1.81MB	70.34%
Student: ResNet20 (AB [38])	~41.21M	~0.27M	~1.81MB	69.47%
Student: ResNet20 (FT [24])	~41.21M	~0.27M	~1.81MB	69.84%
Student: ResNet20 (FSP [27])	~41.21M	~0.27M	~1.81MB	69.95%
Student: ResNet20 (NST [32])	~41.21M	~0.27M	~1.81MB	69.60%
Student: ResNet20 (MFKD)	~41.21M	~0.27M	~1.81MB	**70.88%**

### • Ablation Study

It is known that the location of feature extraction and the way of fusion are two important factors affecting MFKD. Here, we conducted a detailed ablation experiment.

#### a) Extract Location

Thanks to the VGG network having 5 different layer groups, it is convenient for us to explore the influence of the change of the extraction position on MFKD. Therefore the experiment in the ablation study is completed by the VGG network.

Compared with the original extraction combination extracted the output features of the 2nd, the 3rd, and the 4th layer group of the network, we designed two extraction combinations named Combination A and Combination B in what follows. Combination A: we use the output features of the 5th layer group to replace the output features of the 2nd layer group based on the purpose of which is to replace the previous layer features with the bottom layer features. Combination B: we replace the output features of the 3rd layer group with the output features of the 5th layer group owing to the reason that replacing the middle layer features with the bottom layer features. Taking VGG13 as the teacher and VGG8 as the student, the experimental results on CIFAR-100 are shown in Table 6.

**Table 6:** The performance of the student network VGG8 on the CIFAR-100 dataset by using different extraction combinations.

Original	Combination A	Combination B
73.71%	73.16%	72.73%

It can be seen that the original extraction combination includes the features of the front, the middle and the bottom part of the network, and the performance of MFKD decreases after the front or the middle layer features are missing.

#### b) Fusion Method

We explore the fusion of two feature maps: ADD and CONCAT where ADD refers to adding feature map A and feature map B by means of element-wise, while CONCAT means to concatenate feature map A and feature map B into a new feature map.

When using ADD for fusion, the value of the fusion feature is obtained by averaging the value computed by adding the two feature maps on each pixel. When using CONCAT, a structure such as 1×1conv-3×3conv-3×3conv is applied to process the spliced feature maps to achieve the purpose of dimensionality raising/lowering and feature collection. The result looks like this.

It follows from Tables 7 and 8 that CONCAT performs better than ADD for the residual network ResNet, while ADD is better than CONCAT for the linear network VGG. The choice of fusion method varies with the network structure.

**Table 7:** The Top-1 accuracy of the student network ResNet20 using different fusion methods where the teacher is ResNet56 on CIFAR-100.

Teacher	Student	ADD	CONCAT
72.34%	69.06%	70.18%	70.88%

**Table 8:** The Top-1 accuracy of the student network VGG8 using different fusion methods where the teacher is VGG13 on CIFAR-100.

Teacher	Student	ADD	CONCAT
74.64%	70.36%	73.71%	72.76%

### • Extension

In addition, we perform our method between teacher networks and student networks with different structural styles where ResNet50 with four convolutional layer groups as the teacher network, and VGG8 with five convolutional layer groups as the student network. On dataset CIFAR-100, we extract the output feature maps of the 2nd, the 3rd, and the 4th layer group of ResNet50, and extract the output feature maps of the 3rd, the 4th, and the 5th layer group of VGG8, respectively.

The fused features of ResNet50 are processed by using 1×1 convolutions to match the number of channels with those of VGG8. MFKD improves the Top-1 accuracy of VGG8 by 2.18% compared to regular training. The experimental results are given in Table 9.

**Table 9:** Top-1 accuracy of student network VGG8 on CIFAR-100 dataset.

Model	FLOPS	Params	Memory	Top-1 Accuracy
Teacher: ResNet50	~1.3G	~23.71M	~31.20MB	79.34%
Student: VGG8 (beaseline)	~96.44M	~3.96M	~0.61MB	70.36%
Student: VGG8 (Fitnets [15])	~96.44M	~3.96M	~0.61MB	70.69%
Student: VGG8 (AT [26])	~96.44M	~3.96M	~0.61MB	71.84%
Student: VGG8 (CCKD [34])	~96.44M	~3.96M	~0.61MB	70.25%
Student: VGG8 (VID [33])	~96.44M	~3.96M	~0.61MB	70.30%
Student: VGG8 (RKD [35])	~96.44M	~3.96M	~0.61MB	71.50%
Student: VGG8 (AB [38])	~96.44M	~3.96M	~0.61MB	70.65%
Student: VGG8 (FT [24])	~96.44M	~3.96M	~0.61MB	70.29%
Student: VGG8 (NST [32])	~96.44M	~3.96M	~0.61MB	71.28%
Student: VGG8 (MFKD)	~96.44M	~3.96M	~0.61MB	**72.54%**

## Conclusions and future work

In the present study, the multi-layer feature fusion knowledge distillation (MFKD) is proposed to improve the performance of student network. Specifically, we first design the feature fusion pyramid to effectively fuse multiple layers of features together. Then, the quality of feature maps is refined by the attention mechanism. Finally, by setting hyperparameters, students can choose the object of study to further improve the distillation effect. Experiments show that MFKD can significantly outperform state-of-the-art methods.

In the future, we may explore our MFKD in a comprehensive case of teacher network and student network have different structural styles. Further, the applications of MFKD in image detection, image segmentation and other tasks are another research interests.
